# Editorial: Road trip from mild to severe asthmatic inflammation: the traffic lights of biomarkers in asthma management, volume II

**DOI:** 10.3389/fmed.2025.1642976

**Published:** 2025-06-24

**Authors:** Konstantinos Porpodis, Paschalis Steiropoulos, Spyridon Gougousis, Harissios Vliagoftis, Kalliopi Domvri

**Affiliations:** ^1^Pulmonary Department, Medical School, George Papanikolaou Hospital, Aristotle University of Thessaloniki, Thessaloniki, Greece; ^2^Department of Respiratory Medicine, Medical School, Democritus University of Thrace, University General Hospital Dragana, Alexandroupolis, Greece; ^3^Department of Ear Nose Throat, General Hospital of Thessaloniki “G. Papanikolaou”, Thessaloniki, Greece; ^4^Division of Pulmonary Medicine, Alberta Respiratory Centre, University of Alberta, Edmonton, AB, Canada; ^5^Department of Pathology, General Hospital of Thessaloniki “G. Papanikolaou”, Thessaloniki, Greece

**Keywords:** remission, asthma, biologics, biomarkers, personalized treatment approaches

There is still a clinical need to delineate complex endotypes of asthma and to identify novel biomarkers with high predictive and prognostic value to achieve an optimal personalized approach. This Research Topic, describes current topics in asthma biomarker research, providing a better understanding of the utility of the currently available biomarkers and the current biomarker research regarding asthma remission, and suggesting new approaches to use biomarkers in everyday clinical practice for optimal management of patients with asthma. Our Research Topic assembles six high-quality articles describing the benefits of using biomarkers for asthma management.

Within this context, Chen et al. explored the association between baseline Th2 biomarker levels and clinical manifestations in pediatric asthma and identified predictors of clinical remission. The study included 172 children and the authors evaluated a number of clinical parameters, incuding FeNO, blood eosinophils, and serum biomarkers (TSLP, IL-4/5/13, TARC, Periostin, IgE). The authors concluded that serum TSLP is independently associated with clinical remission in Th2-high pediatric asthma and integration with lung function and IgE may form a composite biomarker panel for remission evaluation. This stratification tool may guide asthma risk stratification and personalized disease management, but longitudinal studies are warranted to validate its prognostic utility.

In a slightly different approach, given the important role of cytokines in asthma pathophysiology, Zheng et al. investigated the causal effects between cytokines and asthma, using the inverse variance weighted Mendelian randomization (MR) method. The MR analysis showed that levels of IL-5 and IL-9 were increased in asthma, indicating the downstream effects of IL-5 and IL-9 on asthma. Besides, they concluded that there was no evidence that cytokines increased or decreased the risk of asthma. Using similar methodology, Zaied et al. investigated the distinct and shared genetic risk factors contributing to the development of unspecified asthma (no age-specific), childhood onset asthma (COA) and adult-onset asthma (AOA). They employed a two-sample MR analysis to elucidate the causal association between genes within lung and whole-blood-specific gene regulatory networks (GRNs) and the development of unspecified asthma, COA, and AOA using the Wald ratio method. They identified genes (including *ORMDL3, PEBP1P3*) whose altered expression in lung or blood is putatively causally associated with unspecified asthma and two age-specific asthma presentations, proposing that the causal genes identified in this analysis hold promise as potential drug targets, emphasizing the need to consider the asthma subtype in the development of asthma drugs.

Xu et al. explored the relationship between the systemic immune-inflammation index (SII) and mortality in patients with asthma. The study included 6,156 participants from the National Health and Nutrition Examination Survey (NHANES) for US adults from 2001 to 2018. Subgroup analyses revealed SII's association with all-cause mortality across various demographics, including age, sex, race, education levels, smoking status, and marital status suggesting that SII may potentially serve as a predictive tool for evaluating asthma mortality rates. Similarly, Tian et al. analyzed data from 40,664 participants from NHANES to assess the relationship between SII and asthma and asthma-related events. They found that SII is positively correlated with the persistence of asthma, yet has limited predictive power for asthma recurrence, highlighting SII's potential as a tool for assessing asthma risk and formulating targeted management strategies. Both studies, by analyzing participants from NHANES revealed the potential use of SII in asthma management.

In a broader population, Porsbjerg et al. aimed to elucidate the association between individual biomarker levels or levels of biomarker combinations before initiation of a biologic with changes in asthma outcomes after therapy with a biologic in real-life. This was a registry-based, cohort study using data from 23 countries, which participate in the International Severe Asthma Registry (May 2017–February 2023); results from 3,751 patients that initiated biologics were included. They concluded that since higher baseline blood eosinophil count, FeNO and their combination can predict biologic-associated lung function improvement, earlier intervention in patients with impaired lung function or at risk of accelerated lung function decline with biologics may be beneficial.

We believe that this Research Topic adds to the current literature and advances our understanding of the role of biomarkers in asthma management given the big cohorts analyzed. It is with great pleasure that we are presenting the articles included in this Research Topic to the asthma research community.

